# Variation in Small Mammal Species Composition and the Occurrence of Parasitic Mites in Two Landscapes in a Scrub Typhus Endemic Region of Western Yunnan Province, China

**DOI:** 10.1002/ece3.72384

**Published:** 2025-10-23

**Authors:** Yun‐Yan Luo, Jia‐Xiang Yin, Zong‐Ti Shao, Zeng‐Kan Liu, Shou‐Qin Yin, Jiang‐Li Lu, Jin‐Chun Li, Rong Wei, Alan Frederick Geater

**Affiliations:** ^1^ School of Public Health Dali University Dali Yunnan Province People's Republic of China; ^2^ Faculty of Medicine, Department of Epidemiology Prince of Songkla University Songkhla Thailand; ^3^ Yunnan Institute of Endemic Disease Control and Prevention Dali Yunnan Province People's Republic of China; ^4^ Baoshan Center for Disease Control and Prevention Baoshan Yunnan Province People's Republic of China; ^5^ Tengchong Center for Disease Control and Prevention Yunnan Province People's Republic of China

**Keywords:** biological characteristics, host species, hurdle negative binomial model, landscape

## Abstract

Western Yunnan Province is a scrub typhus endemic region, providing optimal environments for small mammal hosts and mite vectors. This study explored the association between mite occurrence on small mammals and landscape and host characteristics in this region. Two landscapes, farmland and forest edge, in four locations in Tengchong City were sampled for capturing small mammals and collecting mites. Associations among host, vector, and landscape were analyzed using hurdle negative binomial mixed models. Among 279 small mammals captured, ninety‐five individuals were infested with a total of 8308 mites. Infestation prevalence was higher in farmland than in the forest edge. Mean mite intensity was 87.5/infested mammal. The highest mite intensity was seen in *Neotetracus sinensis* . *Rattus tanezumi* , *Suncus murinus* , *Rattus rattus* , and *Mus pahari* were dominant species. Differences in infestation prevalence and mite intensity were inconsistent across species and landscape. In farmland, *Rattus tanezumi* and *Rattus rattus* had a significantly higher infestation prevalence than non‐dominant species. Adult mammals had significantly higher infestation prevalence independent of species and landscape. Larger body size, longer tail, hind leg, and ear were each related to significantly higher infestation prevalence, but the effects were reduced by half after adjusting for species and landscape. Mite intensity was not related to hosts. In conclusion, infestation prevalence and mite intensity varied across species. Infestation prevalence was higher in farmland than in forest edge and was positively related to adult mammals and larger body dimensions.

## Graphical abstract

Yunnan Province, the most serious scrub typhus endemic areas in China, provides abundant ecological resources that sustain parasite and host populations. The distribution of vector‐borne diseases is driven by interactions between hosts, vectors and the environment, revealing critical ecological dynamics. Variations in parasite abundance across small mammal species highlight the significant role of host‐specific and habitat‐driven factors. Host characteristics and environmental influences emerge as pivotal factors governing the occurrence and severity of parasitic mite infestations, offering insights for disease control strategies.

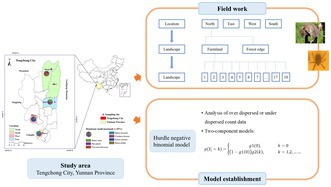

## 1 Introduction

Scrub typhus is a vector‐borne disease in which mites are the main vector and host for transmitting and preserving the bacterial agent *Orientia tsutsugamushi* (*Ot*) (Bahk et al. 2021). There are over 3000 species of mites worldwide, with more than 510 species having been reported in China. Notably, approximately 50% of these species have been recorded in Yunnan Province, southwestern China (Ding et al. 2021). Around 50 species are known to carry pathogens such as *Ot*, Hantavirus and *Bartonella* (Chaisiri et al. 2023; Guo et al. 2023). While *Leptotrombidium delicense* (*L. delicense*) and *L. scutellare* are the main vectors responsible for transmitting *Ot* in China (Luo and Yin 2019), a broader spectrum of mite species, including *Eutrombicula*, *Walchia*, *Gahrliepia* and *Helenicula* etc., has also been identified as carriers of *Ot* around the world (Alkathiry et al. 2022; Elliott et al. 2019; Kim et al. 2020). Small mammals are the reservoir host, with host selection of mites in natural ecosystems encompassing over 30 species across the orders *Rodentia*, *Eulipotyphla*, and *Scandentia*. Furthermore, *Mustela kathiah* of the order *Carnivora* has also been reported to be infested with mites (Lv et al. 2018), highlighting the expansive nature of this parasitic relationship. Low host specificity may potentially increase the probability of mite transfer between hosts and enhance the likelihood of encounters with humans (Huang et al. 2017; Gebrezgiher et al. 2023). In scrub typhus endemic areas, a high mite density may increase the probability of *Ot* transmission within the small mammal community. Additionally, higher mite abundance could lead to more frequent or intense parasitic behavior, potentially increasing the risk of mite bites not only in small mammals but also in humans (Peng et al. 2018). Once *Ot* infection spills over from wildlife to humans, it poses an increased health burden to residents. While *Ot* infection in small mammals or mites provides direct evidence for disease profiles in nature, the abundance of small mammals and mites can serve as proxies for scrub typhus risk (Obiegala et al. 2021). Previous studies have reported a positive correlation between the mite index (mite abundance as described below) and *Ot* infection; increasing populations of small mammals and mites can facilitate the occurrence and transmission of *Ot* in endemic areas (Elliott et al. 2021).

Host‐associated and environment‐associated factors are crucial limiting factors that control the occurrence and infestation of parasitic mites within a host population (Smith et al. 2023). This is because of the complex life cycle of mite, which includes free‐living stages in soil or leaves, as well as those feeding on vertebrate hosts (Wei et al. 2020). Previous studies showed that host‐associated factors, such as body size, sex with hormone levels, and reproductive activities, play significant roles in determining the infestation conditions of mites in small mammals (Smith et al. 2023). Larger hosts, increased hormone levels, or periods of reproductive activity may facilitate the susceptibility of the host to harbor mites (Alkathiry et al. 2022). Moreover, environmental factors also play a crucial role in the host‐vector‐pathogen relationship. Warmer temperature and more humid conditions promote the survival and reproduction of mites, indirectly increasing the risk of *Ot* infection spread to non‐endemic areas (Lu et al. 2021). Landscapes such as shrubland and agricultural fields play a significant role in sustaining mite populations, indirectly fostering the transmission of *Ot* in nature (Alkathiry et al. 2022; Chaisiri et al. 2017; Li et al. 2023). We consider that the abundance of mites in small mammals and the diversity of small mammals may be a driver of scrub typhus spread in nature, perhaps by facilitating the transmission and preservation of *Ot* in endemic areas. Clarifying the host‐vector‐environment association quantitatively can facilitate an understanding of the occurrence and transmission mechanism of *Ot* in nature and thereby inform more effective strategies for preventing zoonotic spillover to humans.

Since 1948, Yunnan Province has become one of the most serious scrub typhus endemic areas in China. In recent decades, around 50% of recorded scrub typhus cases were from western Yunnan. Tengchong City, administratively at the county level, had an increasing trend of scrub typhus incidence unlike other counties in western Yunnan Province. During the period 2010 to 2019, the incidence varied from 8.99 to 31.76/100,000 person‐years; the highest incidence was 37/100,000 person‐years reported in 2018. Small mammals can play a proxy role in public health surveillance programs; understanding the ecology and epidemiology of small mammals and vectors in scrub typhus endemic areas can signal potential risks when *Ot* infections spill over from wildlife to humans (Elliott et al. 2021; Obiegala et al. 2021). However, only a few studies have investigated the parasitic mites on specific small mammal species in Yunnan province. Comprehensive knowledge of interaction among the host, vector, and environment is inadequate in areas with high scrub typhus incidence. Hence, this study aimed to explore how host biological characteristics and landscape type influence the occurrence of parasitic mites in small mammals, assessing differences in the infestation prevalence and intensity across small mammal species between two landscape types and examining the associations between host traits and mite infestation patterns. The study hypothesized that both mite prevalence and intensity would be positively associated with landscape type and host species. Specifically, the study hypothesized that farmland—where food and shelter are more abundant—parasitic mites would exhibit a stronger response to host characteristics compared to forest edge. Additionally, certain host traits, such as larger body size or older age, would be harbor higher levels of mite infestation than smaller or younger individuals.

## 2 Materials and Methods

### 2.1 Study Setting

**FIGURE 1:**
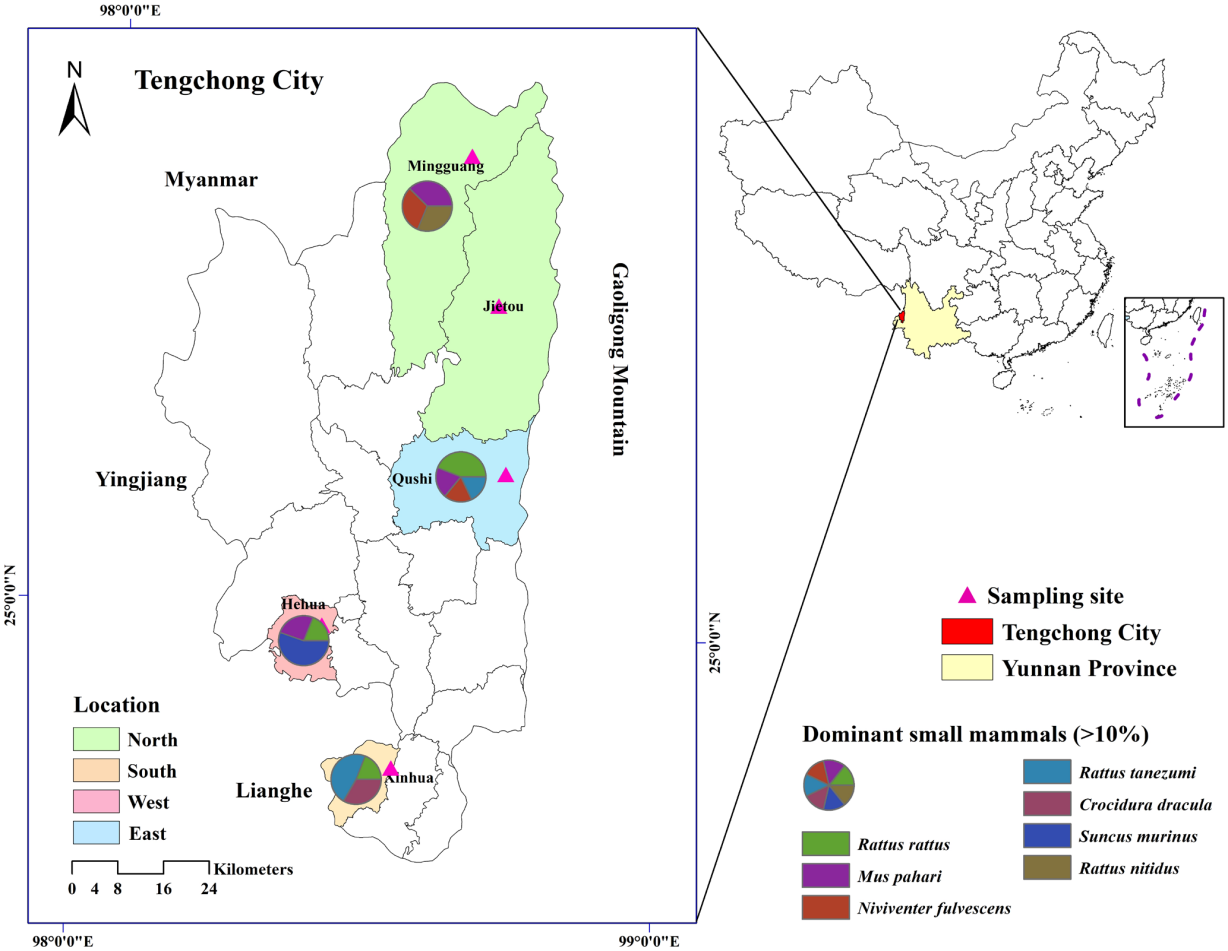
Sampling sites across different locations in Tengchong city. The map was created for an illustrative sample location only.

A cross‐sectional study for the abundance of small mammals and parasitic mites was conducted in July 2022 in Tengchong City, western Yunnan Province. July is generally the month when the incidence of scrub typhus peaks in Yunnan Province. Tengchong City (E 98°05′ to 98°45′, N 24°38′ to 25°52′) is located at the foot of Gaoligong Mountain having diverse small mammal species and an appropriate ecology environment for mite development (Shown in Figure 1) (Guo et al. 2023). Furthermore, it is near the Myanmar border, which is one of the important China‐Myanmar boundary lines providing convenience to residents or travelers for living and commercial activities. Within Tengchong City, there is a gradient of elevation, with the lowest in the south and the highest in the north, ranging from 930 m to 3780 m. In 2019, the total population was about 673,700 (60.5% of the population inhabited rural areas) (Yunnan_Population_Statistics 2019).

Farmland and forest edge were selected as landscape types of interest, as both are settings where small mammals and humans co‐occur, providing opportunities for mite–human contact and potential pathogen transmission. Farmland refers to areas primarily used for agricultural activities such as crop cultivation or orchards, typically characterized by human management and simplified vegetation structure. Forest edge represents a transitional zone between forest interiors and adjacent open habitats, supporting a mixture of species from both ecosystems and facilitating greater interactions among hosts, vectors, and humans.

### 2.2 Sample Size Considerations

The main aim of the study was to describe the occurrence of mites in terms of the proportion of captured small mammals having mite infestation (“infestation prevalence”, IP) and the number of mites per infested mammal (“infestation intensity or mite intensity”, MI). As the values of neither of these parameters was known beforehand, the sample size (number of small mammals in each location/landscape combination) was estimated for a range of possible prevalence and intensity values that would yield sufficiently precise estimates. The sample size for estimation was documented in Table S1.

Finally, the sampling was designed to obtain at least 30 captured mammals per location/landscape combination. It was estimated that around 10% of traps placed overnight would yield a captured small mammal. Thus, at least 300 traps per location/landscape combination needed to be placed overnight to obtain this number of mammals. To maximize the chance of traps capturing a mammal, the 300 traps were distributed in each location/landscape combination among approximately 18 subsites.

### 2.3 Small Mammal and Ectoparasite Mite Collections

#### 2.3.1 Small Mammals Captured

Four locations in Tengchong City were selected, with two principal landscapes (farmland and forest edge) chosen at each location, including the North location (N 25°30′19.1160″, E 98°40′12″), East location (25°14′10.2480″, 98°36′53.2692″), West location (24°59′14.1108″, 98°24′17.7192″) and South location (24°45′23.0000″, 98°28′59.0000″) (Figure 1). Between 18 and 21 subsites were selected to place mousetraps (15.5 cm × 8.5 cm) in each landscape of each location, with at least 25 traps placed in a linear layout at each subsite (totally at least 300 traps). In each landscape at each location, the traps were placed in the afternoon of one day and collected early the following morning. All traps used peanuts as bait.

Small mammals were removed from mousetraps and placed individually in separate bags before being transferred to the lab. A cotton ball soaked with ether was placed in each bag with the small mammals to anesthetize ectoparasites. The small mammals were then identified by species and screened for ectoparasites.

#### 2.3.2 Small Mammal Identification

Small mammals were identified according to the guidelines for the classification of mammals (Zheng et al. 2008) and the key morphology characteristics were recorded (i.e., body size, tail, hind leg and ear length). Sex was identified according to anogenital distance, visible evidence such as descended testes in adult males or enlarged mammae in adult females. Age (adult or juvenile) was estimated based on external traits like weight, tail length, and the color of fur (Ponnusamy et al. 2022).

#### 2.3.3 Ectoparasitic Mite Collection

The pelage of small mammals was examined, with emphasis on the ears, feet, abdomen, and genitals (Ponnusamy et al. 2022). Embedded mites appeared in clusters as red or orange spots and were removed using sterile pointed tweezers from their point of contact with the small mammal's skin under a stereo microscope and 40× microscopes, then the number of mites was counted using a stereo microscope (see Figure S1). Among them, 10%–20% of the total mites harvested from each small mammal were immediately transferred into 1.5 mL Eppendorf tubes with 75% ethanol for further identification, and the rest were preserved in 1.5 mL Eppendorf tubes (average 50 mites as one pool) with absolute ethanol for later molecular analysis (Seo et al. 2021; Ponnusamy et al. 2022). The mite samples were all stored at 4°C in the laboratory.

### 2.4 Statistical Analysis

The distribution and composition of small mammals were summarized using descriptive statistical methods. Small mammal species, whose individuals accounted for at least 10% of all mammals captured overall were considered to be the dominant species (Migliorini et al. 2002). Due to the non‐normal distribution of species characteristics measured as continuous variables, those characteristics were dichotomized based on being “>” or “<=” median. The occurrence of parasitic mites in small mammals was illustrated by the infestation prevalence of small mammals (the percentage of small mammals infested with mites in captured small mammals, IP) and mite intensity (the mean number of mites per infested mammal, MI) (Obiegala et al. 2021). The mean number of mites per individual mammal within species (MA) was quantitatively illustrated in a scatter plot under R 4.3.0 software. The definition details are described below (Obiegala et al. 2021; Bush et al. 1997; Chen et al. 2023):

1. The occurrence of parasitic mites in small mammals:

Infestation prevalence (IP) = (number of small mammals with infested mites/total number of small mammals captured) *100 (%).

Mite intensity (MI) = total number of mites collected/number of small mammals infested with mites.

2 The mean mite abundance:

Mean mite abundance (MA) = total number of mites collected/total number of small mammals captured.

In this study, the outcome variable was the occurrence of parasitic mites in small mammals (including IP and MI). The independent variables were species, landscape and location of small mammals captured, and biological characteristics of small mammals (sex, age, body size, tail, hind leg, and ear length). As the outcome variable was counting data with a high proportion of zeros (65.9%, 184/279), first, the overdispersion was assessed by comparing the simulated residual and observed residual under the fitted model, and the observed and expected residuals were plotted as quantile‐quantile (QQ) plots, and within‐group deviations of different independent levels were compared by the Levene test for homogeneity of variance. Second, zero‐inflation was assessed by comparing the expected distribution of zeros against the observed values (Obiegala et al. 2021). Then, the factors influencing the parasitic mite occurrence in small mammals were explored using Hurdle negative binomial mixed‐effects regression models, in which location was considered to be the random element due to repeated observations.

Hurdle negative binomial (HNB) mixed‐effects regression models were constructed and visualized by “pscl”, “glmmTMB”, and “sjPlot” packages in R version 4.3.0. This HNB mixed‐effects regression model can effectively deal with the data having an excess of zeros and an over‐dispersed distribution, enabling evaluation of the effects of host traits and landscape type on mite occurrence in small mammals. There are two components in the regression model: the first part modeling the infestation prevalence odds (number of infected mammals/number of non‐infected small mammals) in a logistic (binary) model, and the second part modeling the intensity among those individual mammals that were infested based on a truncated negative binomial distribution. Model selection criteria were not applied, as the objective of this study was not to identify the most parsimonious model but to evaluate the effects of specific covariates across a series of models, as below (Tables 1, 3). To ensure robustness, collinearity in each model was assessed using Variance Inflation Factors (VIF) calculated with the “car” package; the fit of each model was checked by residual analysis using the “DHARMa” package (Feng 2021; F 2017) or Likelihood‐Ratio Test (LR test). The prevalence odds ratio (OR) and intensity ratio (IR) were estimated.

**TABLE 1:** The details of hurdle negative binomial mixed effects modeling.

Type of model	Variables	Modeling
Univariate model I	Sex; Age; Body size; Tail length; Hind leg length; and Ear length	Number of mites ~ Sex|Age|Body size|Tail length|Hindleg length|Ear length
Adjusted model II	Adjusted by Sex and Age	Number of mites ~ Body size + Sex + Age
	Adjusted by Sex, Age, and Body size	Number of mites ~ Tail length|Hindleg length|Ear length + Sex + Age + Body size
Adjusted model III	Adjusted by Sex|Age and the species‐landscape combinations	Number of mites ~ Sex + Age + Species×Landscape
	Adjusted by Sex, Age, Body size, and the species‐landscape combinations	Number of mites ~ Tail length|Hindleg length|Ear length + Sex + Age + Body size + Species×Landscape

To comprehensively analyze the key host and environmental factors affecting the infestation prevalence and mean intensity, two levels of regression models were constructed. (1) At the first level, a model was developed to estimate the overall associations of species and landscape with the occurrence of parasitic mites in small mammals. Adjusted predictions of mite abundance for species‐landscape combinations were generated based on the fitted model. (2) At the second level, individual mammal characteristics were examined, first in separate univariate models, and then after controlling for sex, age, body size, and the species‐landscape combinations, and finally in a combined model. In each level analysis, the HNB model partitioned the response into two components. First, a binary model estimated the probability of infestation (presence or absence of mites). Once truncated to exclude non‐infested individuals, a count model was fitted to estimate mite intensity among infested hosts. This two‐part framework captures distinct biological processes governing infestation occurrence and severity. The same set of predictors (host species, landscape type, and biological traits) was included in both the logistic (binary) and count (truncated negative binomial) components of the HNB model to ensure comparability between the processes determining infestation occurrence and intensity. The modeling details are shown in Table 1 and Table 3.

## 3 Results

### 3.1 Diversity Small Mammal Species Collected

A total of 3269 traps were placed (1735 in the farmland and 1534 in the forest edge); 3209 traps, or 98.2% of all traps placed were recovered. Totally 279 small mammals were captured across two landscapes in Tengchong City 132 in the farmland and 147 in the forest edge, and comprised 23 diverse species (4 orders, including 6 species of *Eulipotyphla*, 15 species of *Rodentia*, 1 species of *Scandentia*, and 1 species of *Carnivora*) (Table 2). The geographic distribution of the dominant small mammal species (> 10%) is shown in Figure 1. Of the captured small mammals, 11.1% (31/279) were collected in the north location of Tengchong City, 24.4% in the east location, 39.1% in the west location, and the rest (25.4%) in the south location (see Table S2).

**TABLE 2:** The distribution of small mammals and mite prevalence from different landscapes in Tengchong City, western Yunnan Province, China^a^.

Species	Farmland			Forest edge			Total		
	No. Infested/Captured small mammals (%)	No. mite	Mite abundance	No. Infested/Captured small mammals (%)	No. mite	Mite abundance	No. Infested/Captured small mammals (%)	No. mite	Mite abundance
*Rattus tanezumi*	18/25 (72.0)	1409	56.36	8/24 (33.3)	925	38.54	26/49 (53.1)	2334	47.63
*Suncus murinus*	2/11 (18.2)	76	6.91	4/37 (10.8)	474	12.81	6/48 (12.5)	550	11.46
*Rattus rattus*	17/22 (77.3)	1878	85.36	11/25 (44.0)	1400	56.00	28/47 (59.6)	3278	69.74
*Mus pahari*	1/7 (14.3)	34	4.86	5/25 (20.0)	144	5.76	6/32 (18.8)	178	5.56
*Crocidura dracula*	4/11 (36.4)	184	16.73	4/8 (50.0)	241	30.13	8/19 (42.1)	425	22.37
*Niviventer fulvescens*	3/11 (27.3)	104	9.45	2/6 (33.3)	31	5.17	5/17 (29.4)	135	7.94
*Rattus nitidus*	3/8 (37.5)	57	7.13	2/2 (100.0)	144	72.00	5/10 (50.0)	201	20.10
*Anourosorex squamipes*	2/8 (25.0)	17	2.13	0/2 (0.0)	—	—	2/10 (20.0)	17	1.70
*Neotetracus sinensis*	4/4 (100.0)	824	206.00	0/3 (0.0)	—	—	4/7 (57.1)	824	117.71
*Eothenomys eleusis*	0/3 (0.0)	—	—	0/3 (0.0)	—	—	0/6 (0.0)	—	—
*Eothenomys miletus*	0/4 (0.0)	—	—	1/2 (50.0)	100	50.00	1/6 (16.7)	100	16.67
*Apodemus draco*	0/4 (0.0)	—	—				0/4 (0.0)	—	—
*Crocidura attenuata*	0/2 (0.0)	—	—	0/2 (0.0)	—	—	0/4 (0.0)	—	—
*Dacnomys millardi*	—	—	—	0/3 (0.0)	—	—	0/3 (0.0)	—	—
*Eothenomys cachinus*	0/2 (0.0)	—	—	0/1 (0.0)	—	—	0/3 (0.0)	—	—
*Leopoldamys edwardsi*	0/1 (0.0)	—	—	1/2 (50.0)	93	46.50	1/3 (33.3)	93	31.00
*Micromys minutus*	0/3 (0.0)	—	—				0/3 (0.0)	—	—
*Niviventer confucianus*	2/2 (100.0)	87	43.50				2/2 (100.0)	87	43.50
*Tupaia belangeri*	0/2 (0.0)	—	—				0/2 (0.0)	—	—
*Apodemus chevrieri*				0/1 (0.0)	—	—	0/1 (0.0)	—	—
*Bandicota indica*	1/1 (100.0)	86	86.00				1/1 (100.0)	86	86.00
*Crocidura suaveolens*	0/1 (0.0)	—	—				0/1 (0.0)	—	—
*Melogale moschata*				0/1 (0.0)	—	—	0/1 (0.0)	—	—
Total	57/132 (43.2)	4756	45.06	38/147 (25.9)	3552	24.16	95/279 (34.1)	8308	29.78

^a^ Small mammal species arranged based on the number of captured; Blank represents that this species wasn't captured; Hyphen symbol represents that no parasitic mites were collected due to mammal weren't infested with mites.

Among captured small mammals, species accounting for more than 10% of individuals were *Rattus tanezumi* (17.6%), *Suncus murinus* (17.2%), *Rattus rattus* (16.8%), and *Mus pahari* (11.5%) (Table 2). The two most common species overall, *Rattus tanezumi* and *Rattus rattus* , were among the dominant species in both landscapes, accounting for 18.9% and 16.7% in the farmland, and 16.3% and 17.0% in the forest edge, respectively. In addition, *Suncus murinus* and *Mus pahari* were among the dominant species in the forest edge but not in the farmland (Table 2). Species accounting for more than 10% of individuals differed somewhat across locations (see Table S2), *Rattus tanezumi* and *Rattus rattus* were dominant species in all locations except the north; *Mus pahari* and *Niviventer fulvescens* were dominant in the north and east locations. Other species which were among the dominant species in specific locations were *Rattus nitidus* (north), *Suncus murinus* (west), and *Crocudura dracula* (south) (see Table S2).

### 3.2 Infestation Prevalence and Mite Abundance by Species of Mammal

Overall, 95 small mammals were infested with parasitic mites, giving an overall infestation prevalence of 34.1% (95/279). The infestation prevalence of small mammals was obviously higher in the farmland (43.2%) than in the forest edge (25.9%) (Table 2). Among species for which more than two individuals were captured, *Rattus rattus* (59.6%) had the highest infestation prevalence among small mammals, followed by *Rattus tanezumi* (53.1%) and *Crocidura dracula* (42.1%). Although *Neotetracus sinensis* had a higher infestation prevalence (57.1%), the number of individuals captured was too small to be representative (Table 2).

**FIGURE 2:**
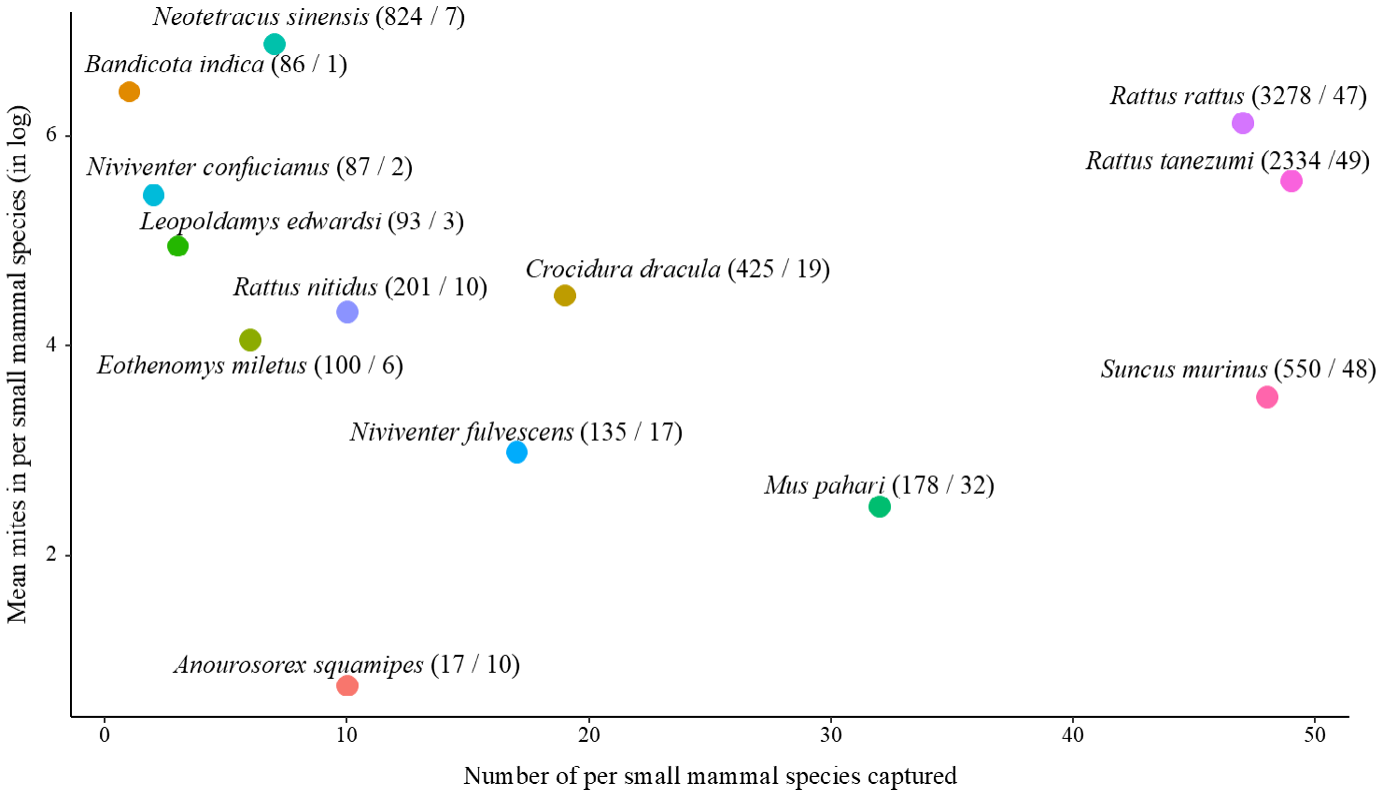
Dynamics distribution of small mammal species and mean mite abundance on small mammal species. The Y‐axis represented the log base 2‐transformed mean number of mites per small mammal species captured (mean mite abundance per small mammal species).

A total of 8308 parasitic mites were collected from small mammals, the overall mean mite abundance was 29.8 (8308/279), and the mite intensity was 87.5 (8308/95). The mean mite abundance of each small mammal species is shown in Figure 2 (The mean mite abundance on the y‐axis was transformed to base‐2 logarithm), and the specific number of mites per small mammal species was shown in Figure S2. Thirteen small mammal species were infested with parasitic mites, with mean mite abundance ranging from 1.7 to 117.7 per individual among the small mammal species. The species with the highest mean mite abundance was *Neotetracus sinensis* , which had a mean of 117.7 mites per individual. *Rattus rattus* , *Rattus tanezumi,* and *Suncus murinus* also had high numbers of mites per individual species, with 69.9, 47.6, and 11.5, respectively; these species also had higher capture numbers compared to other species (Figure 2).

### 3.3 Infestation Prevalence and Intensity by Species and Landscape

The dominant species and landscape of captured small mammals were explored to determine the overall associations with the occurrence of mites in small mammals (Table 3). There was a wide diversity in infestation prevalence across species‐landscape combinations, ranging from *Mus pahari* at 14.3% to *Rattus rattus* at 77.3% in the farmland, and from 10.8% in *Suncus murinus* to 44.0% in *Rattus rattus* in the forest edge. *Rattus tanezumi* and *Rattus rattus* exhibited high infestation prevalences in the farmland, with moderately high prevalences in the forest edge. *Suncus murinus* showed moderate prevalence in the farmland but low prevalence in the forest edge. *Mus pahari* had an intermediate prevalence in both the farmland and the forest edge.

**TABLE 3:** The effect of species‐landscape combinations on the occurrence of mites in small mammals.

Predictors	Observed prevalence (%)	Prevalence odds ratio*	95% CI	LR test	*P* ^#^
Infestation prevalence model					
Intercept		0.40	0.23–0.67		
Species/Landscape*				< 0.01	
Others/Farmland	28.40	1 (Ref.)^b^			—
Others/Forest edge	27.80	0.97^bc^	0.39–2.38		0.950
*Rattus tanezumi* /Farmland	72.00	6.67^a^	2.33–16.67		—
*Rattus tanezumi* /Forest edge	33.30	1.27^b^	0.47–3.45		0.008
*Suncus murinus* /Farmland	18.20	0.56^bc^	0.11–2.86		—
*Suncus murinus* /Forest edge	10.80	0.31^c^	0.10–0.98		0.521
*Rattus rattus* /Farmland	77.30	8.33^a^	2.78–25.00		—
*Rattus rattus* /Forest edge	44.00	2.00^b^	0.77–5.26		0.024
*Mus pahari* /Farmland	14.30	0.42^bc^	0.05–3.70		—
*Mus pahari* /Forest edge	20.00	0.63^bc^	0.21–1.92		0.733

Predictors	Observed mean	Intensity ratio*	95% CI	LR test	*P* ^#^
Intensity part					
Intercept		68.13	36.98–125.52		
Species/Landscape*				0.50	
Others/Farmland	71.52	1 (Ref.)^ab^			—
Others/Forest edge	60.90	0.73^ab^	0.26–2.07		0.557
*Rattus tanezumi* /Farmland	78.28	0.96^ab^	0.38–2.39		—
*Rattus tanezumi* /Forest edge	115.63	1.37^ab^	0.43–4.39		0.484
*Suncus murinus* /Farmland	38.00	0.52^ab^	0.09–3.03		—
*Suncus murinus* /Forest edge	118.50	1.41^ab^	0.34–5.89		0.363
*Rattus rattus* /Farmland	110.47	1.56^b^	0.68–3.54		—
*Rattus rattus* /Forest edge	127.27	1.98^b^	0.74–5.32		0.623
*Mus pahari* /Farmland	34.00	0.46^ab^	0.04–5.25		—
*Mus pahari* /Forest edge	28.80	0.42^a^	0.12–1.55		0.948
Random effects					
*σ* ^2^		0.91			
*τ*_00 location1_		0.05			
ICC		0.05			
*N*_location1_		4			

* Intensity ratios or prevalence odds ratios without common superscripts (a,b,c) within a variable and model are significantly different at *p* < 0.05 by Wald's test. Other species: Non‐dominant species combine in one group.

^#^ *P* values indicate that the differences between farmland and forest edge within each species.

Mite intensity ranged from 28.8 in *Mus pahari* to 127.27 in *Rattus rattus* . *Rattus rattus* had the highest mite intensity per infested individual in both farmland and forest edge, whereas *Mus pahari* had the lowest mite intensity in both landscapes (Table 3).

#### 3.3.1 Modeling the Effects of Species and Landscape on Infestation Prevalence and Intensity

**FIGURE 3:**
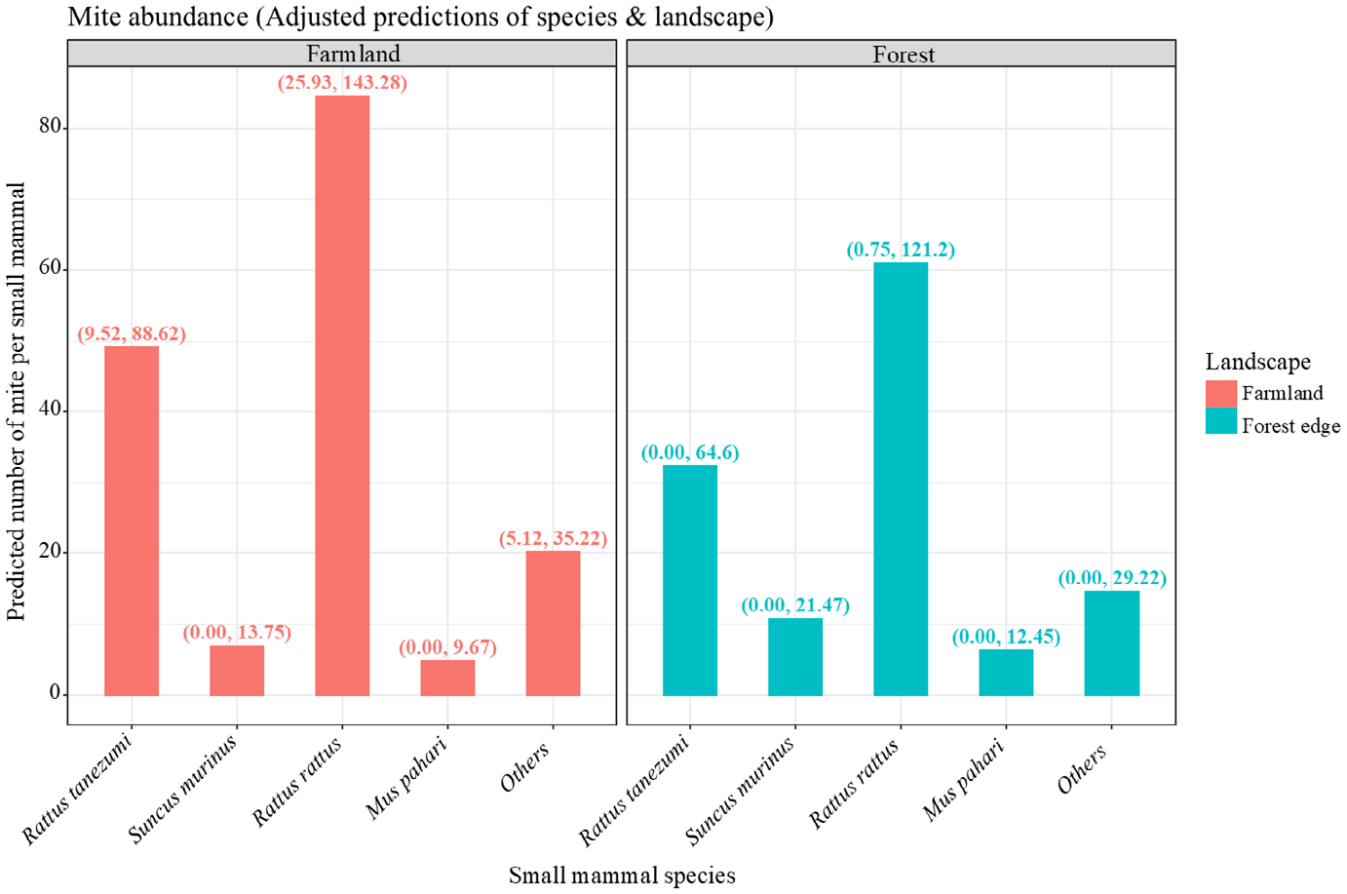
Adjusted Prediction by species and landscape for the number of mites in the small mammals. The confidence interval was shown in brackets.

Using a hurdle negative binomial mixed‐effects model, in which the non‐dominant species (called “other species” in Table 3) in the farmland group was set as the reference, the adjusted prediction for species‐landscape combinations of the occurrence of parasitic mites is reported in Figure 3. In the farmland, the infestation prevalence of *Rattus rattus* and *Rattus tanezumi* was significantly higher than that among the combined non‐dominant species. Among the dominant species, *Rattus tanezumi* and *Rattus rattus* had significantly higher infestation prevalence odds in the farmland than in the forest edge. *Suncus murinus* and *Mus pahari* showed non‐significant differences in mite infestation between farmland and forest edge within each species (Table 3).

The mite intensity in *Rattus tanezumi* , *Suncus murinus* , and *Rattus rattus* was non‐significantly higher in the forest edge compared to the farmland. *Mus pahari* showed a low intensity in the two landscapes with a non‐significant difference (Table 3).

### 3.4 Infestation Prevalence and Intensity by Specific Characteristics of Host

In the univariate modeling, male and adult mammals had a higher infestation prevalence odds compared to female and juvenile although the relationship was not statistically significant, however, after adjusting for the species‐landscape combinations, the odds of infestation prevalence significantly increased 3.34 times in adult mammals compared to juvenile (Table 4). In model II, small mammals with larger body size (> 127 mm), longer tail (> 96 mm), longer hind leg (> 24 mm), or longer ear (> 17 mm) individually had a higher infestation prevalence within each group. For mite intensity, there was a non‐significant increase or decrease effect after adjusting for sex and age to body size, and the other three variables adjusted for sex, age, and body size. Furthermore, in model III, the effects of longer tail, longer hindleg or longer ear were reduced by half after adjusting for sex, age, body size and the species‐landscape combinations (Table 4). In terms of mite intensity, there was no significant differences in the specific characteristics after adjustments (Table 4).

**TABLE 4:** The effect of specific characteristics of the host on the occurrence of mites in small mammals.

Variables	Infestation prevalence part						Intensity part					
	Model I		Model II^a^		Model III^b^		Model I		Model II^a^		Model III^b^	
	Unadjusted prevalence odds ratio	95% CI	Adjusted prevalence odds ratio	95% CI	Adjusted prevalence odds ratio	95% CI	Unadjusted intensity ratio	95% CI	Adjusted intensity ratio	95% CI	Adjusted intensity ratio	95% CI
Sex												
Female	1 (Ref.)				1 (Ref.)		1 (Ref.)				1 (Ref.)	
Male	1.37	0.83–2.27			1.67	0.93–3.03	1.03	0.61–1.75			1.08	0.64–1.83
Age												
Juvenile	1 (Ref.)				1 (Ref.)		1 (Ref.)				1 (Ref.)	
Adult	2.44	0.97–6.25			4.34	1.48–12.76	2.66	0.94–7.55			2.7	0.96–7.69
Body size (mm)												
≤ 127	1 (Ref.)		1 (Ref.)		1 (Ref.)		1 (Ref.)		1 (Ref.)		1 (Ref.)	
> 127	4.55	2.63–7.69	4.35	2.56–7.69	2.08	0.99–4.35	1.65	0.93–2.92	1.61	0.91–2.84	1.43	0.60–3.39
Tail length (mm)												
≤ 96	1 (Ref.)		1 (Ref.)		1 (Ref.)		1 (Ref.)		1 (Ref.)		1 (Ref.)	
> 96	5	2.86–8.33	5.26	2.04–12.5	2.86	0.92–9.09	1.15	0.64–2.06	0.44	0.12–1.60	0.2	0.05–0.83
Hind leg length (mm)												
≤ 24	1 (Ref.)		1 (Ref.)		1 (Ref.)		1 (Ref.)		1 (Ref.)		1 (Ref.)	
> 24	5	2.94–9.09	4.17	1.75–10	2.33	0.84–6.25	1.98	1.08–3.63	2.28	0.84–6.23	3.25	0.77–13.77
Ear length (mm)												
≤ 17	1 (Ref.)		1 (Ref.)		1 (Ref.)		1 (Ref.)		1 (Ref.)		1 (Ref.)	
> 17	3.85	2.33–6.67	2.33	1.16–4.76	1.61	0.71–3.70	1.02	0.59–1.75	0.8	0.42–1.50	0.72	0.32–1.60

^a^ Adjusted model II: Body size adjusted for sex and age; Tail‐, hindleg‐, or ear‐length adjusted for the species‐landscape combinations.

^b^ Adjusted model III: Sex and age adjusted for each other and for the species–landscape combinations; body size, tail length, hindleg length, or ear length as for model II but also adjusted for the species–landscape combinations.

## 4 Discussion

Of the 279 small mammals with 23 diverse species captured in Tengchong City, *Rattus tasnezumi*, *Suncus murinus* , *Rattus rattus,* and *Mus pahari* were the dominant species. Thirty‐four percent were infested with mites. *Rattus tanezumi* and *Rattus rattus* had higher prevalence in both farmland and forest edge. *Rattus rattus* exhibited higher mite intensity, while *Rattus tanezumi* showed intermediate mite intensity. Variation in mite infestation prevalence in small mammals was evident across species‐landscape combinations and host characteristics (age, body size, tail, hind leg and ear length) in Tengchong City.

In this study, *Rattus tanezumi* and *Rattus rattus* were the dominant species in Tengchong City of western Yunnan Province, the result is consistent with previous studies showing that the *Rattus* genus predominates in Yunnan Province, especially in south and southwestern areas (Chen et al. 2023; Lv et al. 2021), where the tropical or subtropical climate provides appropriate environments for living and reproduction. Previous studies have indicated that *Rattus tanezumi* is a known main host of mites and can carry various zoonotic pathogens as a commensal species (Ding et al. 2021). These findings suggest that Tenghcong City, as a scrub typhus natural focus, still presents a potential risk for the cycling transmission of *Orientia tsutsugamushi* within the small mammal community. In addition, in the current study, *Suncus murinus* and *Mus pahari* were also dominant species in the forest edge. Previous study indicated that *Suncus murinus* did not depend on food resources from human garbage and was more likely to live in areas with shelters to protect against predators (Nakamoto and Nakanishi 2013). Therefore, forests could provide better shelter habitats for *Suncus murinus* compared to farmland. Species composition among collected mammals differed somewhat between farmland and the forest edge indicating some ecological complexity in this scrub typhus endemic region. The distribution and density of the small mammal community may have a direct effect on the parasitic mites selecting hosts and further on the spread of scrub typhus (Obiegala et al. 2021).

Variations in geography across different regions can influence the composition of dominant small mammal species, subsequently impacting the infestation load of mites (Guo et al. 2023; López‐Pérez et al. 2022). Consequently, this leads to varying conditions of *Ot* infection within small mammal populations across diverse geographical areas. In the current study, the overall infestation prevalence (IP) of 34.1% and mite intensity of 87.5 were close to those reported in another study, in Ruli City of western Yunnan (IP:47.25%, MI:57.45) (Zhou et al. 2022), but lower than the values reported in Malaysia (IP:78%, MI:241) (Alkathiry et al. 2022). The abundance of mites in small mammals can indirectly affect the spread of scrub typhus from small mammals to humans (Obiegala et al. 2021), therefore, a higher mite intensity in diverse small mammal species in present study may be one of the drivers of the high occurrence of scrub typhus in humans. However, this study is preliminary research on the investigation of landscape and host factors on the abundance of mites; further research should investigate the *Ot* infection in nature to comprehensively understand the high occurrence mechanism of scrub typhus in this area.

This study has shown that diverse small mammal species could be infested with mites, which aligns with previous studies showing that mites exhibit low host specificity (Huang et al. 2017; Ding et al. 2021). However, these results also suggest a tendency for mites to preferentially parasitize locally dominant small mammal species.

In particular, *Rattus rattus* , *Rattus tanezumi* and *Mus pahari* are usually identified as main hosts of mite, thus our findings support previous studies showing that rodents were most susceptible to being infested with mites (Samuel et al. 2021; Ding et al. 2021; Peng et al. 2018; Babyesiza et al. 2023).

In addition to these well‐documented hosts, *Rattus nitidus* , *Crocidura Dracula*, and *Neotetracus sinensis* also had a higher mite infestation prevalence, even increasing the number of mites. These findings highlight the need for further investigation into whether the mites associated with these hosts are generalist parasites or exhibit host specificity. In particular, in this study, *Neotetracus sinensis* , had a high mite intensity, whether landscape characteristics or species characteristics are driving factors needs more research.

The further investigation of mite abundance should extend beyond considering only host preference. Recent alterations in land use due to human activities and the impacts of climate change have significantly influenced the abundance and richness of small mammal populations with many hosts becoming commensal (Liu 2021; Shilereyo et al. 2022; Babyesiza et al. 2023). These changes, in turn, may have impacted the host preference of mites, subsequently influencing the transmission dynamics of *Ot* in small mammal populations. Therefore, it is essential to broaden surveillance to encompass diverse small mammal species and their infestation conditions, as they play a crucial role in shaping the mite infestation and the transmission dynamics of *Ot* among small mammal hosts.

The infestation number of mites (mite intensity) often varies even among individuals of the same small mammal species inhabiting different areas (Peng et al. 2018). This variation can be attributed to several factors such as small mammal and mite population size, altitude, habitat, and food resources (Guo et al. 2023). For instance, *Rattus tanezumi* , exhibited obviously higher infestation prevalence and mite intensity (MA and MI) in the present study compared to previous findings including a border range in Yunnan or southwestern China (Chen et al. 2023; Ding et al. 2021). *Eothenomys miletus* , primarily known as a host of Plague, was found to be infected with *Ot* in Jianchuan County of western Yunnan Province in a previous study (Luo et al. 2023). Despite previous findings reporting a high infestation prevalence and mite abundance on *Eothenomys miletus* (Li et al. 2022), this study collected a relatively low number of mites from individuals.

The host species and ecological environments are the important factors determining the susceptibility of small mammal to harbor mites (Smith et al. 2023; Shilereyo et al. 2022; Babyesiza et al. 2023). In the current study, the results of the effect of species‐landscape combinations revealed significant differences in infestation prevalence of small mammals. Small mammals captured in the farmland were prone to be infested with mites compared to those in forest edge. This may be related to Tengchong City having a tropical monsoon climate, which is suitable for the cultivation of food products and thereby attracting mammals seeking food and shelter during food harvest periods around farmland areas. *Rattus tanezumi* and *Rattus rattus* , as commensal rodents commonly inhabiting households and farmland, were dominant in these habitats and served as primary hosts for parasitic mites. Their high abundance and close association with human environments may contribute to increased mite‐human contact, reflecting an amplification effect—where the presence and dominance of highly competent host species in disturbed or human‐modified habitats can elevate vector abundance and the potential for pathogen transmission to humans. In addition, Tengchong City is located near the Gaoligong Mountain, an area characterized by high biodiversity and abundant vegetation, which provides suitable habitat and food resources for a wide variety of small mammal species (Guo et al. 2023; Peng et al. 2018). These ideal ecological environments may foster more interaction among small mammals, leading to a higher exchange of mite populations between different host species (Gebrezgiher et al. 2023). However, the greater abundance of small mammals in forest ecological environments might result in a dilution effect reducing infestation prevalence in per host species (Babyesiza et al. 2023), this could be attributed to the larger and more heterogeneous habitat in forest, which disperses host and mite populations, thereby lowering contact rates and interrupting pathogen transmission dynamics. Therefore, long‐term surveillance and monitoring should be considered in both farmland and forest edge in the high scrub typhus endemic area to better understand these dynamics effectively, where farmers and travelers pursue their activities and thereby are exposed to the risk of being bitten by mites.

Mite infestation prevalence of small mammals appears to be influenced by the age of the hosts. This study showed that adult mammals significantly increased infestation prevalence but did not significantly affect the number of mites in small mammals. Adult mammals have greater mobility, while juvenile have weaker body conditions, making them less attractive to parasites (Benedek et al. 2024). In addition, the effect of host age was strongly influenced by sex; the increased infestation prevalence in adult mammals was associated with elevated hormone levels, especially during reproductive and courtship behaviors (Smith et al. 2023). Furthermore, high testosterone levels coupled with a poor immune response in small mammals could further lead to increased parasite loads (Benedek et al. 2024).

Previous studies have indicated that larger body of host could be a factor favoring increased parasitic mite aggregation (Smith et al. 2023). The current study showed a higher prevalence in small mammals with large body (> 127 mm), longer tail (> 96 mm), longer hindleg (> 24 mm) or longer ear (> 17 mm). These biological characteristics of small mammals are partly age‐related. Adult mammals have a wide range of activities and a large surface area of whole body, which increases the chance of mites assembling (Lindenfors et al. 2007). In the current study, the effects of body size, tail‐, hindleg‐ and ear‐length were separately adjusted for age, but with only slight differences. However, after adjusting for species‐landscape combinations, these effects were reduced by half in terms of mite infestation. Therefore, the infestation prevalence of small mammals could be partly accounted for both the species of small mammals and the landscape in which they were captured. Different species may have varying susceptibilities to mite infestation due to inherent biological differences or behavioral patterns that affect their exposure to mites. In addition, the landscape provides varying ecological conditions that can influence the density and distribution of both hosts and mites. This indicates that both individual morphological traits and environmental factors play significant roles in determining mite infestation prevalence in small mammals.

This study has some limitations. First, this study was conducted in only one county in the high scrub typhus endemic region of western Yunnan Province. Although geographic location was incorporated as a random effect to account for repeated measures, its effect was found to be negligible; thus, interaction effects between location and landscape were not further evaluated. However, the sampling sites encompassed multiple localities representing two major landscape types, which provided sufficient ecological contrast to explore species‐landscape interactions relevant to host–parasite dynamics. Second, as the small mammals retrieved from the mousetraps were already dead, a small number of non‐embedded mites may have detached from the host, resulting in a minor underestimation of mite intensity. However, in this study, the mousetrap placement period was minimized during the fieldwork to avoid the loss of mites. Third, mite infestation in the present study was confirmed and numbers counted, although without comprehensive identification of the mite species. However, following this study, molecular analysis of mite community with *Orientia tsutsugamushi* is being undertaken, but this is as yet not complete. Nevertheless, this study has revealed some aspects of the host‐vector‐environment association, and thereby contributes elementary evidence for the possible mechanisms of the high incidence of human scrub typhus in this endemic region.

## 5 Conclusion

Diverse small mammals were captured in Tengchong City; *Rattus tanezumi* , *Suncus murinus* , *Rattus rattus* , and *Mus pahari* were the dominant species. *Rattus rattus* had the highest infestation prevalence, but *Neotetracus sinensis* had the highest mite intensity. Infestation prevalence was higher in farmland than in forest edge. The occurrence of parasitic mites in small mammals varied over species‐landscape combinations and host characteristics (age, body size, tail, hindleg, and ear length). Adult mammals had a higher infestation prevalence independent of species and landscape. Larger body size and, independent of body size, longer tail, hindleg, or ear length were associated with greater infestation prevalence, but these effects were partly accounted for by the species‐landscape combinations.

## Author Contributions

**Yun‐Yan Luo:** conceptualization (equal), data curation (equal), formal analysis (equal), investigation (equal), methodology (equal), software (equal), visualization (equal), writing – original draft (equal), writing – review and editing (equal). **Jia‐Xiang Yin:** conceptualization (equal), data curation (equal), funding acquisition (equal), investigation (equal), methodology (equal), project administration (equal), supervision (equal), validation (equal), writing – review and editing (equal). **Zong‐Ti Shao:** investigation (equal). **Zeng‐Kan Liu:** investigation (equal). **Shou‐Qin Yin:** investigation (equal). **Jiang‐Li Lu:** investigation (equal). **Jin‐Chun Li:** investigation (equal). **Rong Wei:** investigation (equal). **Alan Frederick Geater:** conceptualization (equal), formal analysis (equal), methodology (equal), software (equal), supervision (equal), validation (equal), visualization (equal), writing – original draft (equal), writing – review and editing (equal).

## Ethics Statement

The study was approved by the Medical Ethics Committee of Dali University (No. MECDU‐201901‐3). All small mammals captured were allowed and not painful.

## Conflicts of Interest

The authors declare no conflicts of interest.

## Supporting information


**Data S1:** ece372384‐sup‐0001‐AppendixS1.xlsx.

**Table S1:** The details of sample size of estimation and Table S1 (Precision of estimation of infestation prevalence and mite intensity of the study) have been shown in this file.

**Figure S1:** The collection mites from small mammals. The picture A and B showed the embedded mites appeared in clusters as red spots in the ear or perihepatic of small mammal; The picture C showed a mite under the 40× microscopes; The pictured showed a mite under the stereo microscope.

**Table S2:** The distribution of small mammals and mite prevalence from different locations in Tengchong City, western Yunnan Province, China.

**Figure S2:** Dynamics distribution of the number of mites in per small mammal species.
**Figure S3:** Dynamics distribution of the number of mites in per dominant and other small mammal species.

## Data Availability

All the required data is uploaded as Supporting Information.
