# Supplementary material for: Variation in Small Mammal Species Composition and the Occurrence of Parasitic Mites in Two Landscapes in a Scrub Typhus Endemic Region of Western Yunnan Province, China
Source: Ecol Evol. 2025 Oct 23;15(10):e72384. doi: 10.1002/ece3.72384 (PMC12547483; doi:10.1002/ece3.72384)
Supplement: Supplementary file 2 — Table S1: The details of sample size of estimation and Table S1 (Precision of estimation of infestation prevalence and mite intensity of the study) have been shown in this file. [file ECE3-15-e72384-s004.docx]

## **Sample size considerations**

The main aim of the study was to describe the occurrence of mites in terms of the proportion of captured small mammals having mite infestation (“infestation prevalence”, IP) and number of mites per infested mammal (“infestation intensity or mite intensity”, MI). As the values of neither of these parameters was known beforehand, the sample size (numbers of small mammals in each location/landscape combination) was estimated for a range possible prevalence and intensity values that would yield sufficiently precise estimates.

The sample size for estimation of a single proportion with a given precision was calculated using the following equation:

$$n=\frac{Z_{\frac{\alpha}{2}}^{2}P\left( 1-P \right)}{d^{2}}$$

where$Z_{\frac{\alpha}{2}}$is the critical value of the standard normal distribution corresponding to the desired confidence level (95%).$P$ is the estimated infestation prevalence among small mammals, and d is the expected margin of error, which is the maximum acceptable difference between the sample prevalence and the true population prevalence. Given an estimated prevalence of infestation of between 30% and 50%, at least 30 mammals per location/landscape combination were required to provide a relative precision (d/P) of no more than 0.54 (Table S1).

To estimate the required sample size for estimation of mean infestation intensity with an adequate precision, the following equation was used, which is based on an assumed Poisson distribution:

$$n=\left( \frac{Z_{\frac{\alpha}{2}}^{2}\lambda}{d^{2}} \right)^{2}$$

where $\lambda$ is the assumed mean number of mites per infested small mammal (MI), d is the expected margin of error, which is the maximum acceptable difference between the sample mean number of mites per infested mammal and the population mean. As infestation intensity can be considered to be a continuous variable, the required sample size to provide an adequate precision was considerably lower than that for estimation the infestation prevalence. For an estimated mean infestation intensity in the range of 5 to 50 mites per infested mammal, at least 10 infested mammals per location/landscape combination would be sufficient to provide a relative precision (d/𝞴) of 0.28, providing the infestation prevalence were to be at least 30%.

Hence, the sampling was designed to obtain at least 30 captured mammals per location/landscape combination. It was estimated that around 10% of traps placed overnight would yield a captured small mammal. Thus, at least 300 traps per location/landscape combination needed to be placed over night to obtain this number of mammals. To maximize the chance of traps capturing a mammal, the 300 traps were distributed in each location/landscape combination among approximately 18 subsites.

Table S1 Precision of estimation of infestation prevalence and mite intensity of the study

| Infestation Prevalence part: ≥ 30 small mammals captured | | | |
| --- | --- | --- | --- |
|  | Prevalence (P) | d | d/P (Relative error) |
|  | 0.2 | 0.14 | 0.70 |
|  | 0.3 | 0.17 | 0.54 |
|  | 0.5 | 0.18 | 0.36 |
| Mite intensity (based on Poisson distribution): ≥ 10 small mammals infested | | | |
|  | Estimated mean intensity (𝞴) | d | d/ 𝞴 (Relative error) |
|  | 5 | 1.4 | 0.28 |
|  | 10 | 2.0 | 0.20 |
|  | 20 | 2.8 | 0.14 |
|  | 50 | 4.4 | 0.09 |
