# Supplementary material for: Variation in Small Mammal Species Composition and the Occurrence of Parasitic Mites in Two Landscapes in a Scrub Typhus Endemic Region of Western Yunnan Province, China
Source: Ecol Evol. 2025 Oct 23;15(10):e72384. doi: 10.1002/ece3.72384 (PMC12547483; doi:10.1002/ece3.72384)
Supplement: Supplementary file 3 — Figure S1: The collection mites from small mammals. The picture A and B showed the embedded mites appeared in clusters as red spots in the ear or perihepatic of small mammal; The picture C showed a mite under the 40× microscopes; The pictured showed a mite under the stereo microscope. [file ECE3-15-e72384-s001.docx]

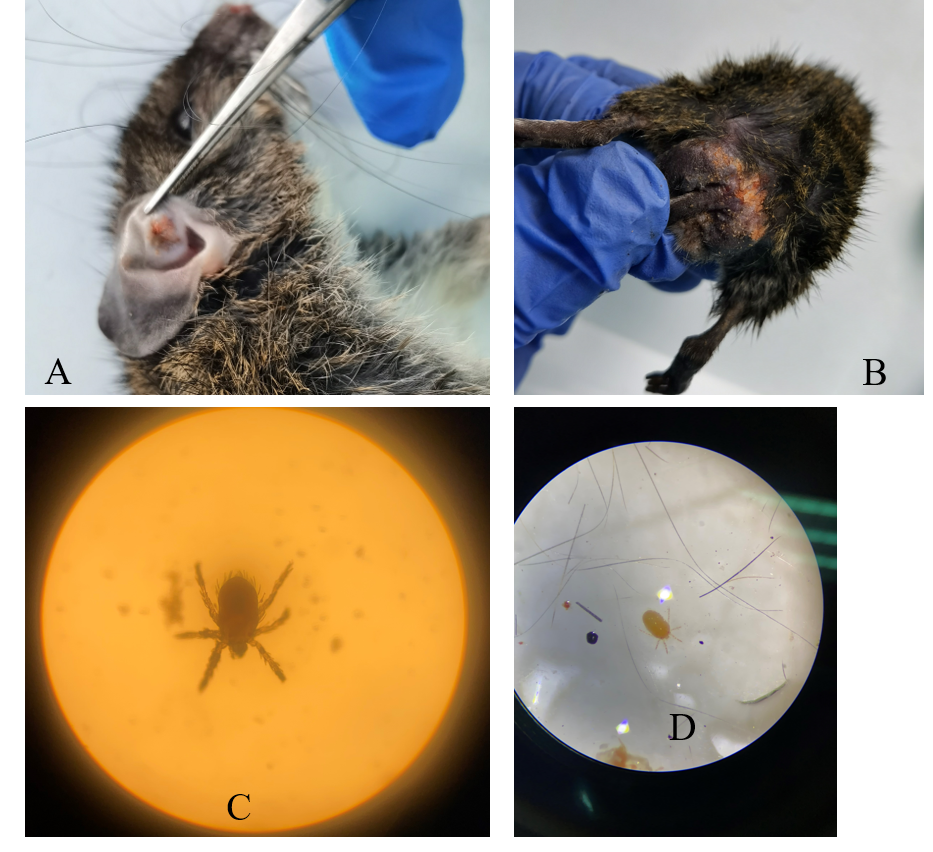


Figure S1 The collection mites from small mammals. The picture A and B showed the embedded mites appeared in clusters as red spots in the ear or perihepatic of small mammal; The picture C showed a mite under the 40× microscopes; The pictured showed a mite under the stereo microscope.
