# Supplementary material for: Variation in Small Mammal Species Composition and the Occurrence of Parasitic Mites in Two Landscapes in a Scrub Typhus Endemic Region of Western Yunnan Province, China
Source: Ecol Evol. 2025 Oct 23;15(10):e72384. doi: 10.1002/ece3.72384 (PMC12547483; doi:10.1002/ece3.72384)
Supplement: Supplementary file 4 — Table S2: The distribution of small mammals and mite prevalence from different locations in Tengchong City, western Yunnan Province, China. [file ECE3-15-e72384-s003.docx]

Table S2 The distribution of small mammals and mite prevalence from different locations in Tengchong City, western Yunnan Province, China^#^

| Small mammal species | North | | East | | West | | South | | Total | |
| --- | --- | --- | --- | --- | --- | --- | --- | --- | --- | --- |
|  | No. Infested / Captured small mammals (%) | No. Mite | No. Infested / Captured small mammals (%) | No. Mite | No. Infested / Captured small mammals (%) | No. Mite | No. Infested / Captured small mammals (%) | No. Mite | No. Infested / Captured small mammals (%) | No. Mite |
| *Rattus tanezumi* | 0/1(0.0) | - | 6/9(66.7) | 128 | 2/19(10.5) | 6 | 18/20(90.0) | 2200 | 26/49(53.1) | 2334 |
| *Suncus murinus* | 0/1(0.0) | - | 0/1(0.0) | - | 2/41(4.9) | 76 | 4/5(80.0) | 474 | 6/48(12.5) | 550 |
| *Rattus rattus* | 1/3(33.3) | 2 | 14/22(63.6) | 1642 | 6/14(42.9) | 260 | 7/8(87.5) | 1374 | 28/47(59.6) | 3278 |
| *Mus pahari* | 2/6(33.3) | 112 | 3/10(30.0) | 32 | 1/9(11.1) | 34 | 0/7(0.0) | - | 6/32(18.8) | 178 |
| *Crocidura dracula* |  |  | 0/4(0.0) | - | 0/1(0.0) | - | 8/14(57.1) | 425 | 8/19(42.1) | 425 |
| *Niviventer fulvescens* | 1/5(20.0) | 1 | 3/9(33.3) | 104 | 0/1(0.0) | - | 1/2(50.0) | 30 | 5/17(29.4) | 135 |
| *Rattus nitidus* | 1/5(20.0) | 7 |  |  | 2/3(66.7) | 50 | 2/2(100.0) | 144 | 5/10(50.0) | 201 |
| *Anourosorex squamipes* | 0/1(0.0) | - | 1/3(33.3) | 9 | 1/3(33.3) | 8 | 0/3(0.0) | - | 2/10(20.0) | 17 |
| *Neotetracus sinensis* |  |  |  |  | 4/7(57.1) | 824 |  |  | 4/7(57.1) | 824 |
| *Eothenomys eleusis* | 0/1(0.0) | - |  |  | 0/1(0.0) | - | 0/4(0.0) | - | 0/6(0.0) | - |
| *Eothenomys miletus* | 0/1(0.0) | - | 0/1(0.0) | - |  |  | 1/4(25.0) | 100 | 1/6(16.7) | 100 |
| *Apodemus draco* | 0/2(0.0) | - |  |  | 0/2(0.0) | - |  |  | 0/4(0.0) | - |
| *Crocidura attenuata* | 0/2(0.0) | - | 0/1(0.0) | - | 0/1(0.0) | - |  |  | 0/4(0.0) | - |

^#^Small mammal species arranged based on the number of captured; Blank represents that this species wasn’t captured; Hyphen symbol represents that no parasitic mites were collected due to mammal weren’t infested with mites.

Table S2 (Continued) The distribution of small mammals and mite prevalence from different locations in Tengchong City, western Yunnan Province, China^#^

| Small mammal species | North | | East | | West | | South | | Total | |
| --- | --- | --- | --- | --- | --- | --- | --- | --- | --- | --- |
|  | No. Infested / Captured small mammals (%) | No. Mite | No. Infested / Captured small mammals (%) | No. Mite | No. Infested / Captured small mammals (%) | No. Mite | No. Infested / Captured small mammals (%) | No. Mite | No. Infested / Captured small mammals (%) | No. Mite |
| *Dacnomys millardi* |  |  | 0/3(0.0) | - |  |  |  |  | 0/3(0.0) | - |
| *Eothenomys cachinus* |  |  |  |  | 0/3(0.0) | - |  |  | 0/3(0.0) | - |
| *Leopoldamys edwardsi* |  |  |  |  | 1/3(33.3) | 93 |  |  | 1/3(33.3) | 93 |
| *Micromys minutus* | 0/2(0.0) | - | 0/1(0.0) | - |  |  |  |  | 0/3(0.0) | - |
| *Niviventer confucianus* |  |  |  |  |  |  | 2/2(100.0) | 87 | 2/2(100.0) | 87 |
| *Tupaia belangeri* |  |  | 0/2(0.0) | - |  |  |  |  | 0/2(0.0) | - |
| *Apodemus chevrieri* |  |  |  |  | 0/1(0.0) | - |  |  | 0/1(0.0) | - |
| *Bandicota indica* |  |  | 1/1(100.0) | 86 |  |  |  |  | 1/1(100.0) | 86 |
| *Crocidura suaveolens* | 0/1(0.0) | - |  |  |  |  |  |  | 0/1(0.0) | - |
| *Melogale moschata* |  |  | 0/1(0.0) | - |  |  |  |  | 0/1(0.0) | - |
| Total | 5/31(16.1) | 122 | 28/68(41.2) | 2001 | 19/109(17.4) | 1351 | 43/71(60.6) | 4834 | 95/279(34.1) | 8308 |

^#^Small mammal species arranged based on the number of captured; Blank represents that this species wasn’t captured; Hyphen symbol represents that no parasitic mites were collected due to mammal weren’t infested with mites.
