# Supplementary material for: Variation in Small Mammal Species Composition and the Occurrence of Parasitic Mites in Two Landscapes in a Scrub Typhus Endemic Region of Western Yunnan Province, China
Source: Ecol Evol. 2025 Oct 23;15(10):e72384. doi: 10.1002/ece3.72384 (PMC12547483; doi:10.1002/ece3.72384)
Supplement: Supplementary file 5 — Figure S2: Dynamics distribution of the number of mites in per small mammal species. Figure S3: Dynamics distribution of the number of mites in per dominant and other small mammal species. [file ECE3-15-e72384-s005.docx]

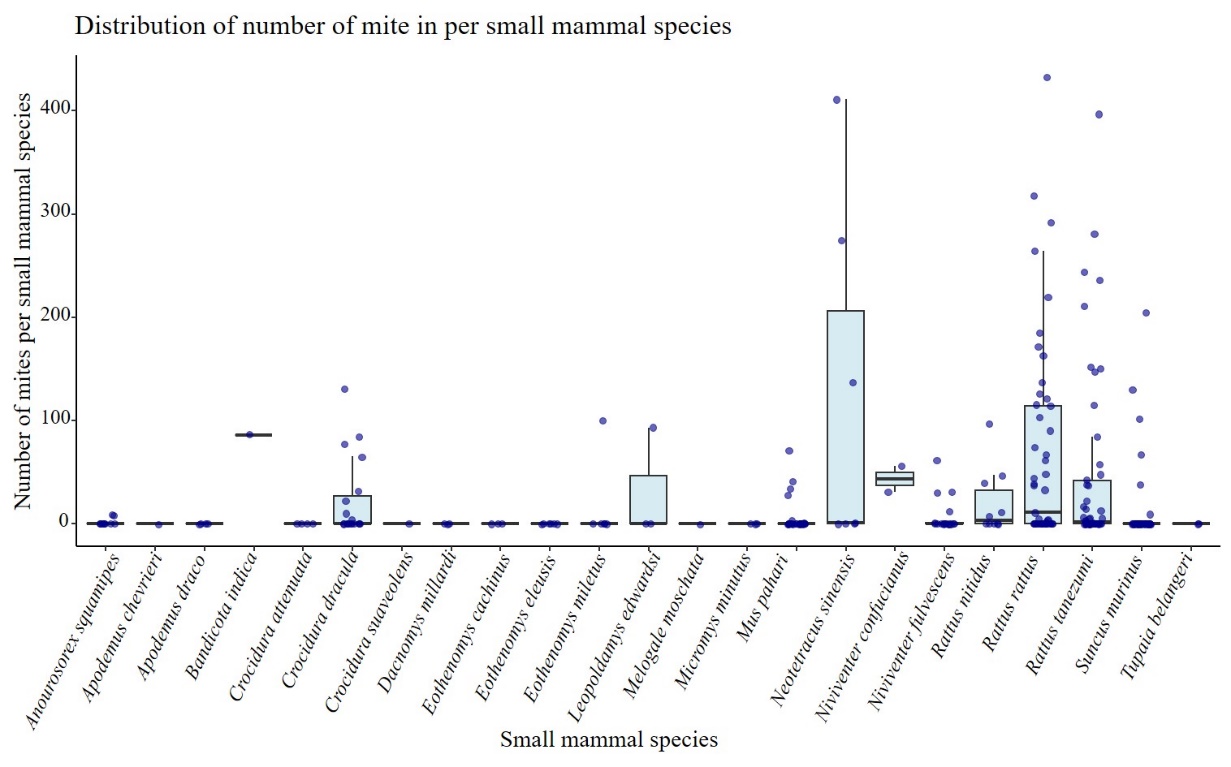


Figure S2 Dynamics distribution of the number of mites in per small mammal species.


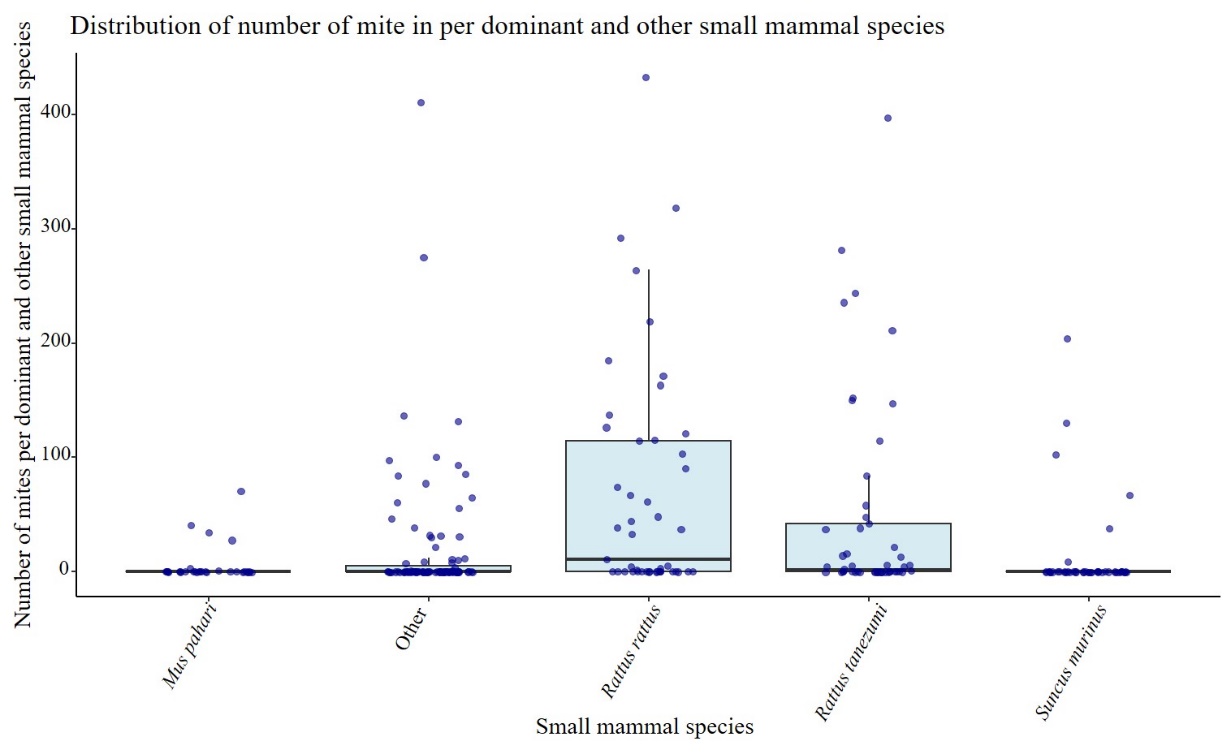


Figure S3 Dynamics distribution of the number of mites in per dominant and other small mammal species.
